# Fourier Ptychographic Microscopic Reconstruction Method Based on Residual Hybrid Attention Network

**DOI:** 10.3390/s23167301

**Published:** 2023-08-21

**Authors:** Jie Li, Jingzi Hao, Xiaoli Wang, Yongshan Wang, Yan Wang, Hao Wang, Xinbo Wang

**Affiliations:** Electrical and Electronic Teaching Center, Electronics Information Engineering College, Changchun University, Changchun 130022, China

**Keywords:** Fourier ptychographic microscopy, spatial attention, channel attention, high-resolution

## Abstract

Fourier ptychographic microscopy (FPM) is a novel technique for computing microimaging that allows imaging of samples such as pathology sections. However, due to the influence of systematic errors and noise, the quality of reconstructed images using FPM is often poor, and the reconstruction efficiency is low. In this paper, a hybrid attention network that combines spatial attention mechanisms with channel attention mechanisms into FPM reconstruction is introduced. Spatial attention can extract fine spatial features and reduce redundant features while, combined with residual channel attention, it adaptively readjusts the hierarchical features to achieve the conversion of low-resolution complex amplitude images to high-resolution ones. The high-resolution images generated by this method can be applied to medical cell recognition, segmentation, classification, and other related studies, providing a better foundation for relevant research.

## 1. Introduction

Traditional optical imaging systems are limited by the spatial bandwidth product (SBP) and require the use of high numerical aperture (NA) objectives to achieve high-resolution (HR) images [1]. However, this comes at the expense of a reduced field of view (FOV). Conversely, the use of low-magnification objectives to maintain a large FOV results in a lower image resolution. Therefore, achieving a balance between these two factors is challenging. In 2013, Fourier ptychographic microscopy (FPM) was proposed as a solution. By using an LED array to illuminate the sample from different angles, FPM captures a set of low-resolution (LR) intensity images containing different frequency domain information. The phase retrieval algorithm is then used to stitch the spectral values of the acquired images together, thus reconstructing an image with both high resolution and a large FOV. FPM can realize large field of view, high-resolution, and quantitative phase imaging [2,3,4,5] at the same time, which has important research value and is widely used in the fields of digital pathology [6,7,8], medical cell detection, and segmentation.

During the acquisition of FPM images, the system is affected by errors and noises, leading to poor image quality for the reconstruction using phase retrieval algorithms. Therefore, many scholars have made improvements to FPM. For example, Tian et al. [9], Lin et al. [10], and Li et al. [11] reduced image acquisition time in the hardware, as well as Jiang et al. [12], Wang et al. [13], Sun et al. [14], and Zhang et al. [15], who utilized untrained neural networks to model the Fourier ptychography imaging process and updated frequency spectrum parameters using backpropagation and gradient descent algorithms for image reconstruction. However, reconstruction using the above methods takes a long time.

To improve the efficiency of image reconstruction, deep learning has been applied to FPM. Using residual learning networks [16,17,18] can accelerate the convergence speed during training and reduce the reconstruction time. Meanwhile, to address the issue of flat images, channel attention mechanisms [19,20,21,22] are used to model the interdependence between feature channels, adaptively rescale the features of each channel, and highlight effective features in the final reconstructed image, improving the fidelity of the reconstruction images. For example, Thanh et al. [23] combined the conditional generative adversarial network (cGAN) framework with a weighted Fourier loss function to effectively learn high-resolution information encoded in the dark field data. Chen et al. [24] proposed a deep learning-based neural network model for FPM, which enhanced the network model’s expressive power and generalization ability while also addressing slow LR image acquisition. However, the imaging process also accumulates various inevitable noises in the measurements. To solve this problem, Zhang et al. proposed the DeUnet method [25] and the FPNN method [26]. The DeUnet method adaptively filters out the noise during the reconstruction process using cross-layer attention mechanisms. The FPNN method uses a synthetic input approach to achieve high-frequency information fusion through the traditional model method, significantly improving FPM’s time resolution. Sun et al. [27] proposed the DFNN method, which splits the network data flow into two branches, allowing simultaneously obtaining HR amplitude and phase information. This model algorithm has good robustness to noise and wave vector bias. Zhang et al. [28] proposed the PbNN-CA reconstruction model, which combines a physics-based network with channel attention modules [19] to simultaneously correct pupil aberration and LED intensity errors while improving noise robustness. Wang et al. [29] proposed the DMFTN method, which uses three networks for multi-scale fusion, and the feature information is fully extracted, which effectively improves image quality and reduces reconstruction time.

To further improve the image reconstruction quality of FPM, this paper integrates the models used in deep learning image super-resolution methods into the FPM network. Inspired by Kim et al. [30], residual networks are applied to enhance network stability, and faster convergence can be achieved during training. The shallow feature output map and the deep feature map can be fused using multiple residual learning in the network to obtain more information-rich images. In addition, spatial attention mechanisms and channel attention mechanisms are added to each residual block to better reconstruct the details of the image.

## 2. Proposed Methods

### 2.1. Network Architecture

**Figure 1:**
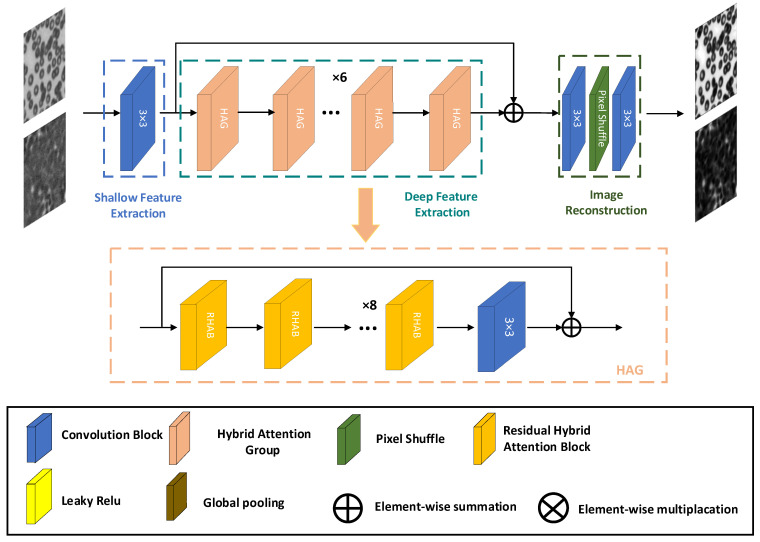
RHAN network architecture diagram.

In this paper, a residual hybrid attention network (RHAN) Fourier ptychographic microscopy reconstruction model is proposed to enhance the quality and speed of image reconstruction. The RHAN consists of three parts, as shown in Figure 1. Shallow feature extraction is composed of a 3 × 3 convolution, which extracts low-level features through convolution operation, retains texture and local detail information, and reduces overfitting risk. The information flow from shallow feature extraction enters deep feature extraction as input. Deep feature extraction is composed of a hybrid attention group (HAG), which provides higher-level semantic information. Each HAG includes multiple residual attention modules composed of channel attention (CA) modules and spatial attention (SA). The high-frequency information after deep feature extraction and the low-frequency information after shallow feature extraction are connected through a residual method to make the image contain richer semantic information. The reconstruction module is composed of sub-pixel convolution and two 3 × 3 convolutions to reconstruct high-frequency information.

### 2.2. Hybrid Attention Group

As shown in Figure 1, the HAG is composed of residual attention modules in series, and a 3 × 3 convolution is introduced afterwards to further extract useful features, thus improving the performance of the model. The HAGs are also connected by residuals, which bring the output information of the previous group into the next group to learn together, effectively enhancing the semantic information and avoiding loss of information during the learning process.

### 2.3. Residual Hybrid Attention Block

**Figure 2:**
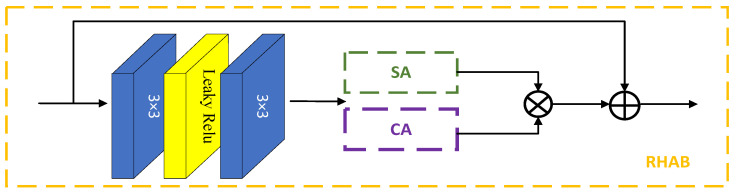
RHAB network architecture diagram.

Inspired by RCAB [20], the channel attention module has been improved as shown in Figure 2. First, the input information flows into a convolution module composed of a 3 × 3 convolution and Leaky ReLU activation function to enhance the feature expression ability of the model. Then, SA is introduced into the CA to facilitate deep feature learning. Unlike RCAB, SA [31] is added to extract fine spatial features and, combined with the CA, to adaptively adjust hierarchical features, thereby enhancing the feature extraction and prediction capabilities of the network.

### 2.4. Spatial Attention

**Figure 3:**
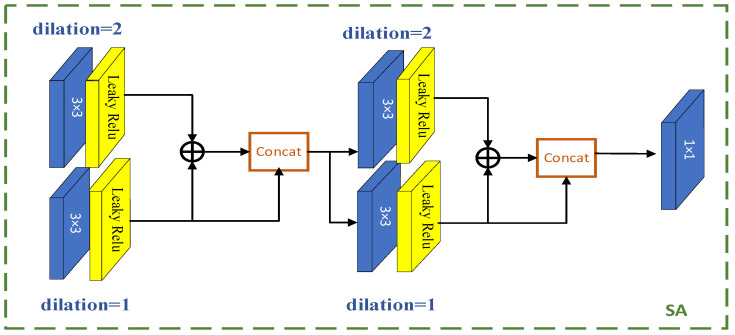
SA network architecture diagram.

In order to improve visual effects and increase receptive fields, dilated convolutional layers with different dilation factors are used to extract multi-scale features instead of using different sizes of convolutional kernels (3 × 3 and 5 × 5). Conducting multi-scale convolutional operations to increase the receptive fields will also increase the network computation, as shown in Figure 3. By using dilated operations with different step sizes, the network can increase the receptive fields without increasing complexity, thus enhancing visual effects. Information fusion is performed after different dilated convolutional operations, which increases the number of channels. Introducing 1 × 1 convolution can make the input and output channel numbers the same.

### 2.5. Channel Attention

**Figure 4:**
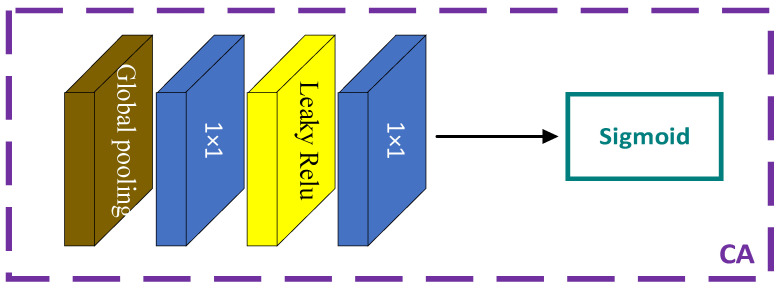
CA network architecture diagram.

As shown in Figure 4, the CA mechanism first downsamples the features through global average pooling, reducing the size to 1 × 1 × C. Different from RCAB [20], it uses the Leaky Relu activation function instead of the Relu activation function and then obtains different weight coefficients through the upsampling operation and sigmoid activation function. The original residual connections are replaced with SA, where important information extracted by the SA is given larger weight, highlighting the required important features to a greater extent. This significantly improves the accuracy of Fourier ptychographic microscopy reconstruction.

## 3. Experimental Results

### 3.1. Data Setting

The experiment used a simulated dataset consisting of 25,600 sets of HR image data. Each set contains two images representing the intensity and phase channels. First, the HR intensity and phase images in each set are combined to generate complex amplitude data. The Fourier ptychographic imaging model is then used to simulate the complex amplitude data. During the simulation imaging process, Gaussian noise with a mean of 0 and a standard deviation of 3 × 10^−4^ is added to simulate the system error noise generated during the actual imaging processes, which serves as the Fourier ptychographic LR data. The traditional Fourier ptychographic reconstruction algorithm, which only iterates once, is used to synthesize the LR complex amplitudes. This results in 25,600 sets of LR input intensity and phase data, with the 25,600 sets of HR image data serving as the ground truth images. The simulation and preprocessing of the dataset are implemented in MATLAB.

The objective lens of the FPM imaging system with a numerical aperture (NA) of 0.13 is used to collect images in the experiment. The light intensity image is recorded by a 2560 × 2560 pixel (6.5 um pixel size) scientific CMOS camera. A planar array is used as a 13 × 13 programmable light source element LED, and the illumination wavelength is 505 nm at 100 mm below the sample to provide illumination. The implementation of the method in this paper was done in Python 3.8 using the PyTorch open-source framework. The evaluation metrics used were peak signal-to-noise ratio (PSNR) and structural similarity (SSIM). To take into account human visual perception, SSIM is used as the loss function. The network was trained with a learning rate of 1 × 10^−4^ using input and ground truth data with a size of 192 × 192 pixels. The network was trained for 200 epochs, with a batch size of 64. The trained model was saved and tested on the experimental platform.

### 3.2. Optimization Algorithm Comparison Experiment

**Figure 5:**
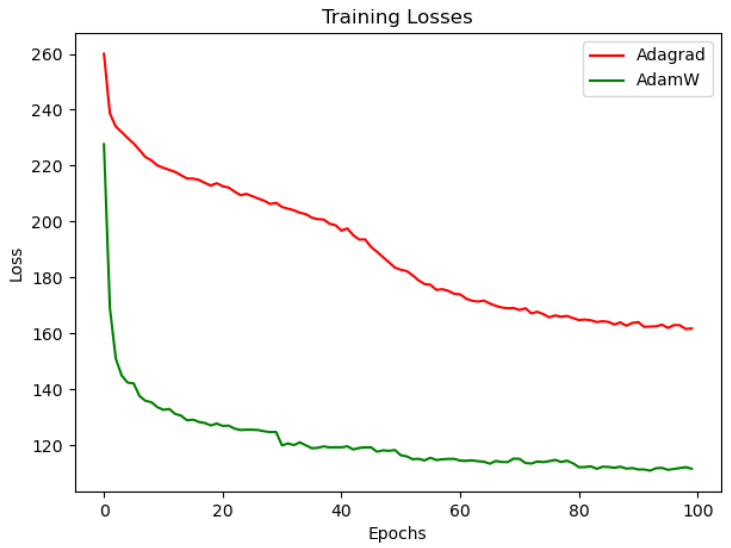
Comparison of the loss function between Adagrad and AdamW optimizers.

To ensure better optimization results, the performance of Adagrad and AdamW optimizers are compared with the same loss and iterations. The curve in Figure 5 shows the loss comparison curve of the two different optimizers during network training. The red curve represents the curve of training the network using the Adagrad optimizer, while the green curve represents the curve of training the network using the AdamW optimizer. The horizontal axis “Epochs” represents the number of training iterations, while “Loss” represents the loss value of each training. PSNR and SSIM are image quality evaluation indexes.

**Figure 6:**
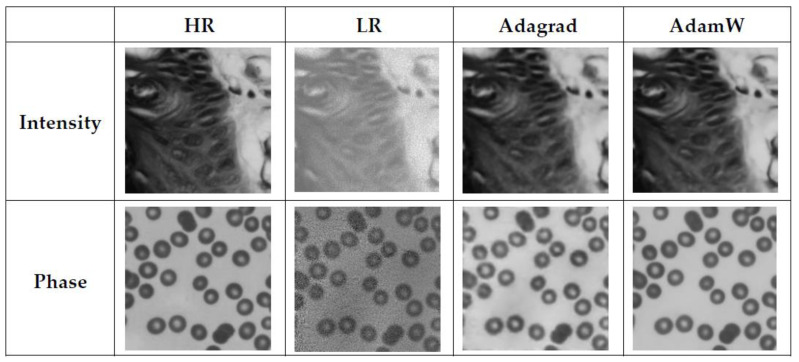
Comparison of the reconstructed results using Adagrad and AdamW optimizers.

The comparison results are shown in Figure 5. The network trained with the AdamW optimizer has a faster reduction of loss and better convergence and is always in a decreasing trend, while the network trained with the Adagrad optimizer has a faster loss reduction in the early stage and an unstable and flattening reduction in the later stage under the influence of the dynamic adjustment of the learning rate. The reconstruction results are shown in Figure 6. The network trained using the AdamW optimizer achieves good results in both reconstructed intensity images compared to the Adagrad method, but the reconstructed phase images are closer to the HR images. As shown in Table 1, the image reconstructed using the AdamW optimizer has higher metrics, which, in summary, demonstrates the effectiveness of the AdamW optimizer-trained network.

### 3.3. Ablation Experiment

Through conducting ablation experiments, the effectiveness of combining spatial and channel attention in Fourier ptychographic reconstruction is validated. In this experiment, RCN represents residual channel attention network, RSN represents residual spatial attention network, and RHAN represents residual hybrid attention network. Under the same conditions of using the same loss, learning rate, noise, etc., the three models were used to train the network separately.

**Figure 7:**
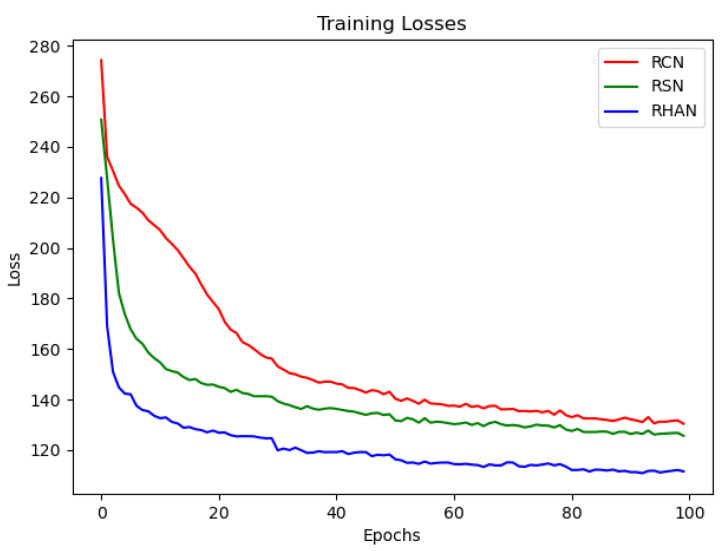
Comparison of the loss curves of RCN, RSN, and RHAN.

**Figure 8:**
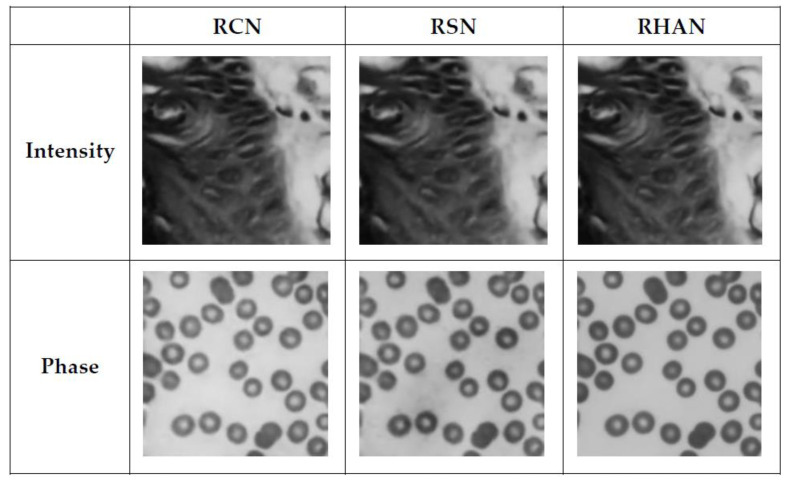
Comparison of the reconstruction results of RCN, RSN, and RHAN.

As shown in Figure 7, all three networks can achieve convergence. Additionally, using the hybrid attention network compared to the other two networks, the loss drops faster and stays in a downward trend. As shown in Figure 8, the intensity images reconstructed using the RHAN model did not change significantly compared to the other two methods, but the phase images were clearer and free of clutter. As shown in Table 2, the image reconstructed using the RHAN model has higher metrics, which, in summary, proves the effectiveness of the RHAN model.

### 3.4. Comparison of Reconstruction Performance under Noise Conditions

**Figure 9:**
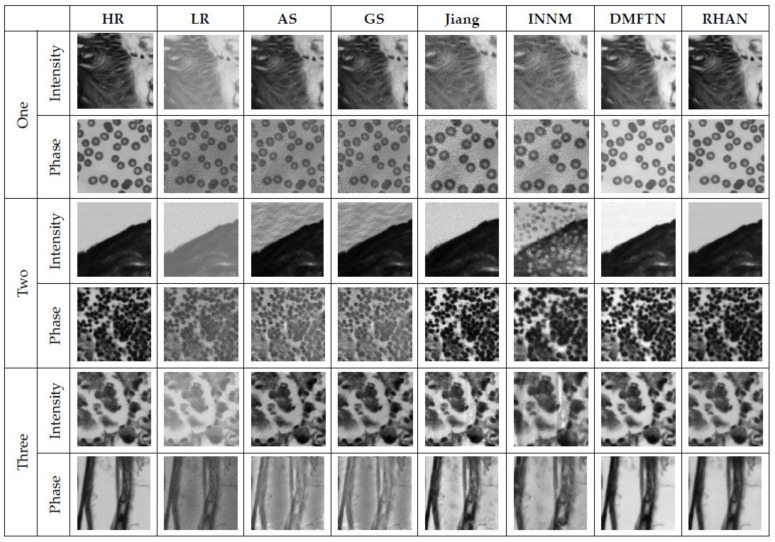
Comparison of the reconstruction results of different images using different methods.

During image acquisition in FPM, Gaussian noise can be introduced due to different brightness of light sources. Therefore, the potential noise effect in the actual data acquisition process is simulated and uses a noise magnitude of 3 × 10^−4^ as the main interference condition for the experiment to verify the robustness and anti-interference ability of the RHAN method. The approach with several other reconstruction methods, including the traditional phase recovery AS method [32], the adaptive step-size reconstruction method GS method [32], the neural network modeling approach proposed by Jiang et al. [12], the INNM reconstruction method based on deep convolutional neural networks [33], the DMFTN method based on multi-scale fusion [29], as well as the RHAN method. The dataset with added Gaussian noise is reconstructed and randomly selected three sets of images from the test set. The reconstruction results of different algorithms under the same noise magnitude (3 × 10^−4^) are shown in Figure 9, and Table 3 shows the corresponding evaluation metrics under different methods.

**Figure 10:**
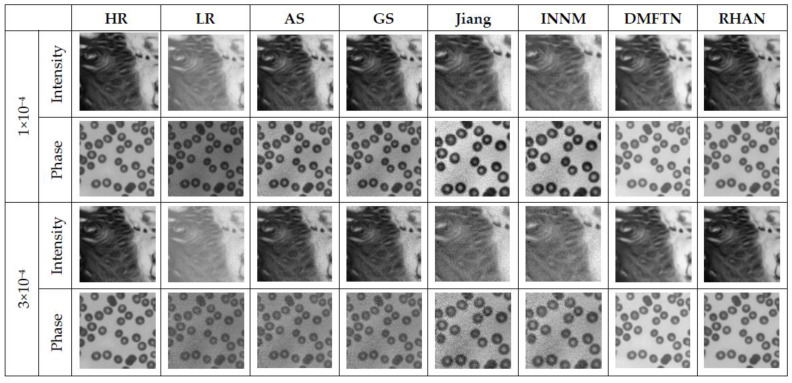
Comparison of reconstruction results of different methods under different noise levels.

During the actual reconstruction process, noise of different levels of magnitude was encountered. In order to verify the universality and robustness to noise of the reconstruction methods, the reconstruction of the same images under different noise conditions were simulated and the reconstruction results and metrics were compared. The levels of noise used in the simulations were 1 × 10^−4^ and 3 × 10^−4^. Figure 10 shows the reconstruction results of the same amplitude and phase image under different noise conditions, and Table 4 shows the corresponding evaluation metrics under different noise conditions.

From the above experimental results, it can be seen that the Fourier ptychographic microscopy reconstruction method using the RHAN has a good reconstruction effect and reconstruction metric values. The amplitude and phase reconstruction images obtained using the DMFTN and RHAN methods are clearer and have smaller errors compared to those obtained using the GS, AS, and INNM methods and are closer to high-resolution images. In Table 3, the bolded results are the optimal ones, and the closer the SSIM metric results are to 1, the better the image reconstruction effect. The RHAN method exhibited good reconstruction performance on different images and noises, indicating strong robustness and generalization capabilities of the network.

### 3.5. Reconstruction Time Comparison on the Real Data Experiment

**Figure 11:**
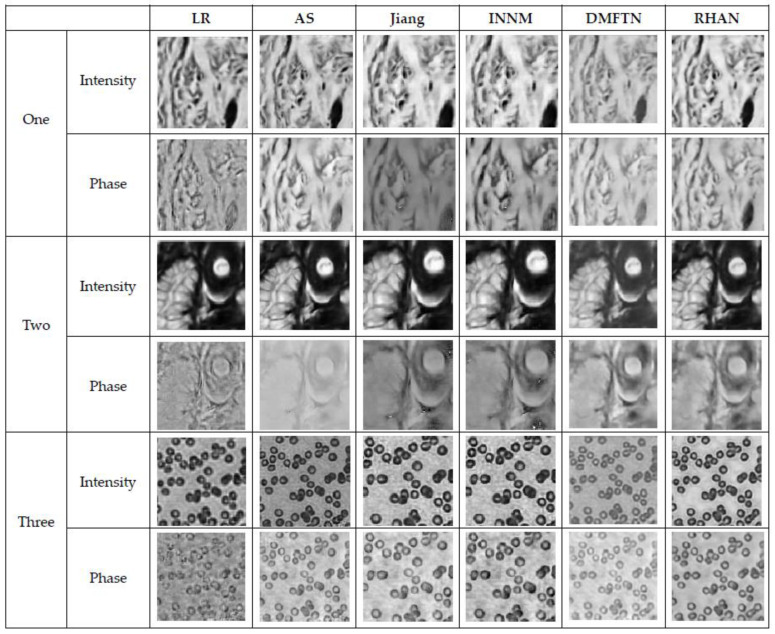
Comparison of reconstruction results of real acquired images.

To further validate the effectiveness of the network, the real collected images were added to the simulated dataset for training the network, and it was tested using low-resolution images acquired from the real dataset. The proposed RHAN method is compared with AS, GS, Jiang, INNM, and DMFTN, and the reconstruction results are shown in Figure 11.

Since the LR images acquired from the system are real, it is not possible to obtain corresponding HR images for evaluation. In addition to comparing the reconstruction results, the reconstruction speed is also an important evaluation metric. In the experiment with real data, the reconstruction time as the evaluation metric is used. Table 5 shows the number of iterations and reconstruction time for each compared method.

From Figure 11, it can be seen that other compared methods are able to reconstruct the rough contours of the image, but the RHAN method can reconstruct the details of the amplitude and phase images better, resulting in clearer texture details compared to other results. According to Table 5, the AS, GS, Jiang, and INNM methods require iterations for reconstruction and have longer reconstruction times. In contrast, the DMFTN and RHAN methods do not need to iterate and have the shortest reconstruction times, achieving the fastest reconstruction speed. 

## 4. Conclusions

This paper proposes a Fourier ptychographic reconstruction method based on a residual hybrid attention network, aiming to address the limitations of traditional phase recovery and reconstruction algorithms. The proposed method combines channel attention and spatial attention based on residual learning to allocate channel weights and extract fine spatial features. By continuously training the network, the method achieves the goal of reconstructing HR images. The simulation results show that by simulating noise in the network training, a model with robustness can be obtained. This solves the problem of high computational cost and poor reconstruction performance of traditional Fourier ptychographic reconstruction algorithms and can be applied to various medical cell reconstructions with strong generalization ability.

## Figures and Tables

**Table 1:** Reconstruction indexes of different optimizers.

RHAN	Adagrad PSNR (dB)/SSIM	AdamW PSNR (dB)/SSIM
Intensity	35.68/0.9371	**36.87/0.9471**
Phase	20.97/0.9265	**25.18/0.9682**

**Table 2:** Reconstruction indexes of different modules.

	RCN PSNR (dB)/SSIM	RSN PSNR (dB)/SSIM	RHAN PSNR (dB)/SSIM
Intensity	36.07/0.9416	36.16/0.9398	**36.87/0.9471**
Phase	17.14/0.9285	23.97/0.9429	**25.18/0.9682**

**Table 3:** The reconstruction metrics of different methods.

		AS PSNR (dB) /SSIM	GS PSNR (dB) /SSIM	Jiang PSNR (dB) /SSIM	INNM PSNR (dB) /SSIM	DMFTN PSNR (dB) /SSIM	RHAN PSNR (dB) /SSIM
One	Intensity	24.77 /0.5192	25.19 /0.5235	16.36 /0.5881	15.30 /0.6200	21.36 /0.8641	**36.87** **/0.9471**
	Phase	18.27 /0.4579	18.46 /0.4415	23.62 /0.7381	24.67 /0.7165	**28.07** /0.9578	25.18 /**0.9682**
Two	Intensity	20.46 /0.6267	17.85 /0.6291	20.19 /0.8460	11.67 /0.5354	29.75 /0.9467	**39.80** **/0.9689**
	Phase	11.36 /0.4993	13.29 /0.5363	23.62 /0.8744	23.91 /0.8684	22.87 /**0.9522**	**28.92** /0.9316
Three	Intensity	21.23 /0.8298	22.40 /0.8418	19.99 /0.8735	18.28 /0.7993	31.31 /0.9316	**35.04** **/0.9592**
	Phase	13.84 /0.6251	13.84 /0.6358	14.09 /0.8076	15.01 /0.8237	24.49 /0.8984	**27.35** **/0.9441**

**Table 4:** Comparison of reconstruction metrics of different methods under different noise levels.

		AS PSNR (dB) /SSIM	GS PSNR (dB) /SSIM	Jiang PSNR (dB) /SSIM	INNM PSNR (dB) /SSIM	DMFTN PSNR (dB) /SSIM	RHAN PSNR (dB) /SSIM
1 × 10^−4^	Intensity	25.91 /0.6816	28.98 /0.6952	23.10 /0.7464	17.18 /0.7190	24.56 /0.9047	**39.66** **/0.9694**
	Phase	21.72 /0.5360	20.35 /0.5346	23.49 /0.7797	26.17 /0.7667	**27.35** /0.9692	23.97 **/0.9709**
3 × 10^−4^	Intensity	24.77 /0.5192	25.19 /0.5235	16.36 /0.5881	15.30 /0.6200	21.36 /0.8641	**36.87** **/0.9471**
	Phase	18.27 /0.4579	18.46 /0.4415	23.62 /0.7381	24.67 /0.7165	**28.07** /0.9578	25.18 /**0.9682**

**Table 5:** Reconstruction indicators of different methods.

Reconstruction Methods	Number of Iterations	Reconstruction Time
AS	50	4.655 s
GS	50	4.132 s
Jiang	50	50 s
INNM	50	500 s
DMFTN	0	0.092 s
RHAN	**0**	**0.075 s**

## Data Availability

Not applicable.
